# Alcohol-Induced Dysregulation of Hydrogen Sulfide Signaling in Alzheimer’s Disease—*Narrative Mechanistic Synthesis Review*

**DOI:** 10.3390/ijms27031595

**Published:** 2026-02-06

**Authors:** Constantin Munteanu, Cristina Popescu, Andreea-Iulia Vlădulescu-Trandafir, Francisco Maraver, José Manuel Carbajo, Gelu Onose

**Affiliations:** 1 Grigore T. Popa University of Medicine and Pharmacy Iași, 700115 Iași, Romania; 2 University of Medicine and Pharmacy “Carol Davila”, 020022 Bucharest, Romania; andreea-iulia.trandafir@drd.umfcd.ro (A.-I.V.-T.); gelu.onose@umfcd.ro (G.O.); 3 Neuromuscular Rehabilitation Clinic Division, Teaching Emergency Hospital “Bagdasar-Arseni”, 041915 Bucharest, Romania; 4 Medical Hydrology Group, Department of Radiology and Rehabilitation, Complutense University of Madrid, 28040 Madrid, Spain; fmaraver@ucm.es (F.M.); jocarbaj@ucm.es (J.M.C.); 5 Professional School of Medical Hydrology, Complutense University of Madrid, 28040 Madrid, Spain

**Keywords:** hydrogen sulfide (H_2_S), Alzheimer’s disease (AD), alcohol consumption, neurodegeneration, oxidative stress, neuroinflammation, mitochondrial dysfunction, homocysteine, gasotransmitters

## Abstract

Alcohol use disorder (AUD) is highly comorbid with psychiatric conditions and is increasingly recognized as a modifiable factor associated with cognitive decline and dementia, including Alzheimer’s disease (AD). While epidemiological and experimental studies consistently demonstrate that chronic alcohol exposure exacerbates neurodegenerative vulnerability rather than implying a single dominant causal pathway, accumulating evidence supports a multifactorial and context-dependent framework in which alcohol acts as a disease-modifying stressor that perturbs endogenous adaptive and resilience mechanisms. Hydrogen sulfide (H_2_S), involved in redox regulation, mitochondrial function, neuroinflammatory control, and vascular homeostasis, has emerged as a candidate pathway that may be indirectly affected by alcohol exposure and relevant to neurodegenerative processes. This narrative mechanistic review synthesizes preclinical and clinical data examining alcohol-induced perturbations and H_2_S-related signaling pathways in the context of AD. We analyzed studies on the effects of acute and chronic alcohol exposure, as well as on cellular processes influenced by H_2_S bioavailability and signaling. Across experimental models and human studies, alcohol exposure was consistently associated with oxidative and mitochondrial stress, neuroinflammation, and vascular dysfunction—processes that overlap with biological domains normally regulated by H_2_S. Alcohol-related cognitive impairment frequently occurs in the absence of proportional increases in classical AD pathology, suggesting that alcohol may accelerate disease progression through non-canonical mechanisms. H_2_S signaling confers resilience against oxidative, inflammatory, and mitochondrial stress, whereas reduced H_2_S bioavailability or disrupted sulfide-dependent signaling increases neuronal vulnerability and cognitive impairment. However, the available data do not support a unidirectional or exclusive role for H_2_S as an integrative driver of alcohol-related AD pathology. H_2_S signaling represents a biologically plausible *convergent and modulatory* pathway linking alcohol exposure to AD risk.

## 1. Introduction

Alzheimer’s disease (AD) and alcohol use disorder (AUD) are major and highly prevalent contributors to the global burden of cognitive impairment and dementia worldwide [1,2], representing a growing medical, social, and economic burden in aging societies [3]. Although distinct clinical and nosological entities, the pathophysiology of AUD and AD is now widely recognized as multifactorial [4], extending beyond the classical hallmarks of amyloid-β plaques and tau neurofibrillary tangles [5]. Increasing attention has therefore shifted toward metabolic, vascular, inflammatory, and redox-related mechanisms that modulate neuronal vulnerability and disease progression [6].

Disease-modifying anti-amyloid therapies such as aducanumab demonstrate that substantial β-amyloid removal can be achieved without uniformly translating into proportional cognitive benefit, underscoring the contribution of parallel, non-amyloid mechanisms to AD progression [7]. These observations reinforce the concept that amyloid pathology alone is insufficient to account for clinical trajectories in AD. Advanced polymer-based delivery systems have been developed to improve the bioavailability and tolerability of symptomatic AD therapies, highlighting the importance of pharmacokinetic optimization in neurodegenerative treatment, where pH-sensitive microspheres composed of poly(vinyl alcohol), alginate, and carboxymethyl cellulose enable the controlled intestinal release of donepezil hydrochloride, reducing gastrointestinal exposure and stabilizing plasma drug levels [8].

Aging-related synaptic dysfunction in AD is increasingly recognized as a critical determinant of cognitive decline [9], often preceding or exceeding the contribution of classical amyloid and tau pathology [10]. In an APP/PS1 mouse model of AD, supplementation with alpha-ketoglutarate or its calcium salt rescued impaired hippocampal long-term potentiation, enhanced synaptic plasticity [11], and restored associative memory mechanisms, effects linked to improved mitochondrial and autophagic [12] function rather than to the direct modulation of amyloid burden [13]. These findings emphasize that neuronal energy metabolism and proteostatic capacity are central determinants of synaptic resilience. These findings highlight the importance of metabolic resilience and bioenergetic support in maintaining synaptic integrity in AD and underscore the potential of metabolic pathways as modulators of neurodegenerative vulnerability [14].

Alcohol is one of the most widely consumed psychoactive substances globally, with patterns of use ranging from low, intermittent intake to chronic heavy consumption and alcohol use disorder [15]. Chronic and heavy alcohol exposure activates neuroimmune signaling pathways and induces mitochondrial dysfunction [16]. Alcohol promotes microglial activation, increases pro-inflammatory cytokine release, and disrupts mitochondrial bioenergetics and dynamics, thereby elevating oxidative stress and impairing neuronal energy homeostasis [17]. These alcohol-induced neuroinflammatory and mitochondrial alterations synergistically exacerbate neurodegenerative vulnerability [18] and accelerate AD disease-related cognitive decline [19], particularly under conditions of sustained or hazardous alcohol use [20].

A substantial body of epidemiological literature has examined the relationship between alcohol consumption and cognitive decline or dementia [21], often reporting non-linear, J-shaped [22], or U-shaped [23] associations. In many observational studies, light-to-moderate alcohol intake has been associated with a lower incidence of dementia [24] compared with abstinence [25]. In contrast, heavy drinking and alcohol use disorder consistently increase the risk of cognitive impairment, early-onset dementia, and mortality. Importantly, these divergent findings do not imply uniform biological effects of alcohol across exposure levels. However, these findings remain highly contested [26].

Methodological limitations—including residual confounding, survivor bias, misclassification of abstainers, competing risk of death, and heterogeneity in exposure assessment—have raised serious concerns about the interpretation of putative protective effects [27]. Mendelian randomization studies [28] and extensive registry-based analyses increasingly suggest that alcohol is unlikely to be intrinsically neuroprotective and instead acts as a disease-modifying stressor whose harmful effects may be obscured under certain epidemiological conditions [29,30,31]. These considerations support the need for mechanistic frameworks capable of integrating epidemiological heterogeneity with biological plausibility.

Alcohol exerts complex and pleiotropic effects on the central nervous system [32]. Chronic ethanol exposure induces dysregulation of protein kinase C (PKC) signaling [33], oxidative and nitrosative stress, mitochondrial dysfunction, excitotoxicity, neuroinflammation, blood–brain barrier disruption, and alterations in cerebral blood flow and white-matter integrity. Neuropathological and biomarker studies have yielded inconsistent evidence that alcohol directly increases amyloid-β or tau pathology in humans. Alcohol-related dementia often lacks the defining histopathological features of AD and may show partial stabilization or reversibility with abstinence. Alcohol, primarily a modifiable risk factor [17], accelerates neurodegeneration through mechanisms that are, at least in part, independent of classical amyloid- and tau-centric pathways. Identifying the molecular systems that mediate this risk remains a key unresolved challenge [34].

Hydrogen sulfide (H_2_S) is an endogenously produced gasotransmitter whose levels and signaling are tightly regulated in the brain [35], as both deficiency and excess can be detrimental [36]. It is synthesized primarily by cystathionine β-synthase, cystathionine γ-lyase, and 3-mercaptopyruvate sulfurtransferase, with distinct cellular localization in neurons and astrocytes. H_2_S exerts neuroprotective effects largely through protein persulfidation, a reversible post-translational modification that preserves protein function under oxidative stress and prevents irreversible cysteine oxidation [37]. Through these mechanisms, H_2_S participates in fine-tuning synaptic transmission, mitochondrial respiration, and neurovascular coupling. In AD, multiple lines of evidence indicate reduced H_2_S bioavailability and diminished global persulfidation, which are associated with impaired synaptic plasticity, mitochondrial dysfunction, increased neuroinflammation, and enhanced tau pathology [38]. Restoration of physiological H_2_S signaling in experimental models improves cognitive performance and attenuates neurodegenerative processes, highlighting the importance of sulfide-dependent signaling for neuronal resilience in AD [39].

H_2_S is identified as a protective metabolic signaling molecule that enhances cellular glucose uptake, mitochondrial efficiency, and resistance to oxidative stress through the activation of PI3K/AKT-dependent pathways [40]. Disruption of H_2_S production, reflected by hyperhomocysteinemia and impaired transsulfuration, is associated with cognitive decline and AD-related pathology [41], whereas restoration of physiological H_2_S signaling in experimental models mitigates amyloid burden, neuroinflammation, and energy deficits in vulnerable brain regions [42].

Importantly, alcohol exposure intersects with virtually every upstream regulator and downstream target of H_2_S signaling. Chronic alcohol use disrupts sulfur amino acid metabolism, promotes hyperhomocysteinemia, depletes antioxidant defenses, impairs mitochondrial function, and induces sustained neuroimmune activation. These processes are precisely those buffered by intact H_2_S signaling under physiological conditions. Yet, despite this striking mechanistic convergence, the role of alcohol-induced dysregulation of H_2_S signaling in AD has not been systematically examined. The existing literature remains fragmented across disciplines, with alcohol research, H_2_S biology, and AD pathophysiology largely developing in parallel rather than in integration.

Several competing and, at times, contradictory hypotheses therefore coexist in the field. One view emphasizes potential cardiovascular or psychosocial benefits of low-dose alcohol exposure, indirectly reducing dementia risk [43]. An opposing view argues that any apparent benefit is artifactual and that alcohol uniformly increases neurodegenerative vulnerability through cumulative metabolic and inflammatory stress. A third, more integrative perspective suggests that alcohol’s effects are conditional, reflecting the balance between protective signaling systems and toxic stressors [44], including endogenous gasotransmitter pathways that support redox and metabolic homeostasis. Resolving these divergent interpretations requires a mechanistic synthesis that moves beyond exposure–outcome associations and focuses on shared molecular pathways.

Patchouli alcohol, a non-ethanol sesquiterpene compound, exhibits marked neuroprotective effects in a sporadic AD animal model by penetrating the blood–brain barrier and attenuating cognitive deficits. Its beneficial actions are mediated through the upregulation of SIRT1, suppression of tau hyperacetylation and hyperphosphorylation, inhibition of neuroinflammatory signaling, and reduction in amyloid-β accumulation, illustrating how modulation of gut microbiota metabolic and redox-regulatory pathways can improve neurodegenerative outcomes independently of classical alcohol exposure [45,46].

**Figure 1:**
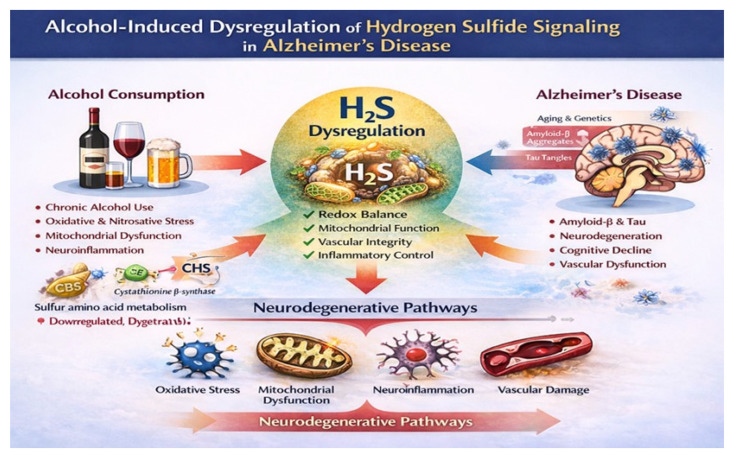
Alcohol-induced dysregulation of hydrogen sulfide (H_2_S) signaling as a convergent mechanism linking alcohol exposure to AD vulnerability. Alcohol exposure is shown to induce oxidative and nitrosative stress, mitochondrial dysfunction, and neuroinflammation through ethanol metabolism and immune activation. H_2_S dysregulation is depicted as a modulatory node rather than a primary causal driver, reflecting its role in maintaining redox balance, mitochondrial efficiency, vascular integrity, and inflammatory control under physiological conditions. Alcohol-induced suppression or imbalance of this signaling axis lowers neuronal resilience, shifting the system toward vulnerability. AD-related outcomes emerge as downstream consequences of this reduced resilience, including enhanced sensitivity to amyloid-β and tau-associated stress, synaptic dysfunction, cerebrovascular impairment, and cognitive decline, often in the absence of proportional increases in classical neuropathological burden. Oxidative stress, mitochondrial failure, neuroinflammation, and vascular damage.

The aim of the present review is to critically examine and integrate the available evidence on alcohol-induced dysregulation of H_2_S signaling in the context of AD (Figure 1). Specifically, we seek to (i) summarize current knowledge on H_2_S signaling in brain physiology and AD, (ii) synthesize experimental, clinical, and epidemiological evidence linking alcohol exposure to pathways that regulate H_2_S metabolism and function, and (iii) evaluate whether disruption of H_2_S signaling provides a unifying mechanistic framework to explain alcohol-related acceleration of neurodegenerative processes. By doing so, this review aims to clarify an underexplored but potentially central biological axis, reconcile conflicting findings in the alcohol–dementia literature, and highlight novel therapeutic and preventive targets relevant to both AD and alcohol-related cognitive decline.

**Figure 2:**
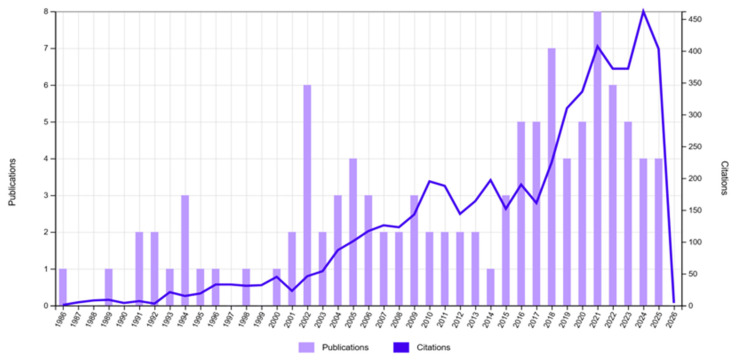
Temporal evolution of publications and citations related to alcohol exposure, H_2_S signaling, and neurodegeneration. The figure illustrates the longitudinal development of the scientific literature on the relationship between alcohol exposure, H_2_S biology, and AD-related mechanisms over the last four decades. Bars represent the annual number of publications, while the overlaid line depicts the corresponding annual citation counts, serving as a proxy for scientific impact and conceptual consolidation. An initial exploratory phase is evident from the late 1980s through the mid-1990s, characterized by sporadic publications and low citation density, reflecting early descriptive and mechanistic work primarily focused on alcohol neurotoxicity and oxidative stress. From the early 2000s onward, a progressive increase in both publications and citations can be observed, coinciding with expanding interest in mitochondrial dysfunction, redox signaling, sulfur amino acid metabolism, and gasotransmitters. A marked acceleration after approximately 2015 highlights a phase of rapid conceptual integration, during which H_2_S signaling, neuroinflammation, neurovascular dysfunction, and systemic metabolic pathways increasingly converged with AD research. The steep rise in citation counts during this period indicates growing recognition of these mechanisms as central, disease-modifying processes rather than peripheral phenomena. The recent plateau and slight fluctuation in publication numbers, accompanied by sustained high citation rates, suggest that the field is maturing, with emphasis shifting from hypothesis generation toward integrative synthesis, translational relevance, and critical re-evaluation of causal frameworks. Overall, the figure supports the interpretation that the alcohol–H_2_S–neurodegeneration axis has evolved from a fragmented research niche into a coherent, high-impact area within AD research (https://www.webofscience.com/ accessed on 9 January 2026).

The body of literature considered for this narrative synthesis spans a broad, methodologically heterogeneous range of studies addressing alcohol exposure, H_2_S biology, and AD-related processes (Figure 2). The evidence base includes epidemiological cohort and case–control investigations, experimental animal and cellular studies, biomarker and neuroimaging analyses, and mechanistic and integrative reviews published over several decades. Collectively, these studies reflect substantial diversity in study populations, definitions of alcohol exposure, biological models, and analytical approaches. A recurrent methodological feature of the literature is that direct quantification of H_2_S signaling—such as measurements of sulfide pools, enzyme activity, or protein persulfidation—is predominantly confined to experimental and preclinical contexts. In contrast, human studies most often interrogate upstream or downstream correlates of H_2_S metabolism, including alterations in homocysteine homeostasis, oxidative and nitrosative stress, mitochondrial dysfunction, and neuroinflammatory pathways, which serve as indirect indicators of disrupted sulfide-dependent signaling in neurodegenerative vulnerability.

## 2. Alcohol Exposure and Alzheimer’s Disease-Related Outcomes

Light-to-moderate alcohol consumption, previously associated with a significantly lower risk of dementia [2,47] compared with non-drinking [48], induces measurable alterations in cerebrospinal fluid biomarkers [49] of AD even in cognitively intact, middle-aged adults [50]. In a cross-sectional analysis, individuals reporting low-to-moderate alcohol intake or high adherence to a Mediterranean alcohol-drinking pattern showed higher amyloid-β positivity and increased Tau/Aβ and phosphorylated Tau/Aβ ratios compared with abstainers, suggesting that alcohol exposure may influence preclinical neurodegenerative processes well before the onset of overt cognitive symptoms [51].

Patients with severe alcohol-related cognitive impairment show a clinical trajectory that differs fundamentally from AD, with evidence of partial reversibility under sustained abstinence and structured cognitive rehabilitation. In contrast to AD patients, individuals with alcohol-related cognitive impairment demonstrated stabilization or improvement in executive functions over follow-up, highlighting that alcohol-related neurocognitive damage reflects a dynamic, modifiable condition rather than a relentlessly progressive neurodegenerative process [52]. This reversibility supports mechanisms dominated by neurotransmitter imbalance, metabolic stress, and neuroinflammatory activation rather than irreversible neuronal loss.

In a patient with long-standing alcohol-related dementia and no typical neuroimaging features of AD, treatment with the acetylcholinesterase inhibitor donepezil was associated with marked improvement in global cognition, executive function, and activities of daily living over a three-month period. This observation supports the concept that alcohol-related cognitive impairment may involve reversible neurotransmitter dysfunction rather than irreversible Alzheimer-type neurodegeneration, highlighting alcohol-related dementia as a distinct and potentially modifiable clinical entity [53].

Heavy alcohol consumption produces cognitive and neurobiological alterations that overlap with, but are not identical to, those observed in AD, including brain atrophy, white-matter damage, and cholinergic dysfunction [54]. Despite clear biological plausibility, epidemiological evidence does not consistently support alcohol as a direct causal factor for AD, suggesting instead that alcohol may influence disease vulnerability, timing of onset, or progression through shared but partially distinct mechanisms [55]. A large post-mortem analysis comparing heavy alcohol consumers with age- and sex-matched controls found no significant differences in amyloid-β deposition, tau neurofibrillary pathology (Braak stage), α-synuclein aggregation, or vascular infarct burden after adjustment for age and sex. Despite clear evidence of liver pathology and alcohol-related brain changes, heavy alcohol consumption did not increase the classical neuropathological hallmarks of AD, supporting the conclusion that alcohol-related dementia follows a distinct, non-amyloidogenic pathogenic pathway [56].

Across epidemiological studies, the association between alcohol exposure and AD-related outcomes becomes clearer when examined in relation to exposure intensity, timing, and population vulnerability. Studies that operationalize alcohol exposure as chronic heavy drinking or alcohol use disorder consistently report substantially elevated dementia risk, with effect sizes often exceeding those of many traditional vascular risk factors. Large administrative and insurance-based cohorts show that individuals with alcohol use disorder have markedly higher incidence of AD and other dementias, frequently accompanied by an earlier age of diagnosis, often by several years, compared with non-exposed populations. These studies also show that the risk is not confined to AD alone but extends to mixed and unspecified dementias, suggesting a broad neurodegenerative vulnerability rather than a pathology-specific effect. Importantly, the strength of association is greatest for early-onset dementia, indicating that alcohol exposure in midlife or earlier adulthood may accelerate neurodegenerative processes long before typical late-life disease onset [57].

Prenatal alcohol exposure produces long-lasting cognitive deficits and induces biochemical changes in the adult brain that resemble key features of AD. In a rat model, fetal alcohol exposure resulted in persistent impairments in spatial learning and memory at 12 months of age, accompanied by increased acetylcholinesterase activity, elevated amyloid-β and phosphorylated tau levels, and upregulation of BACE1 and UNC5C in the cerebral cortex and hippocampus, indicating that early-life alcohol exposure can establish a pro-neurodegenerative molecular milieu later in life [58].

Adolescent binge alcohol exposure induces persistent proinflammatory neuroimmune activation that accelerates AD-associated basal forebrain pathology later in life. Post-mortem human tissue and transgenic mouse models demonstrate that early-life alcohol exposure upregulates HMGB1–RAGE/TLR4 signaling, enhances microglial activation, and promotes the loss of cholinergic neuron markers in the basal forebrain, effects that occur independently of dense amyloid plaque deposition and are partially reversible through the inhibition of HMGB1 signaling [59]. In a triple-transgenic AD mouse model, early-life alcohol exposure increased intraneuronal amyloid-β and phosphorylated tau in the hippocampal and limbic regions and induced durable microglial proinflammatory signaling, effects that were prevented by anti-inflammatory intervention, indicating that immune priming represents a key mechanism linking alcohol exposure to accelerated neurodegenerative vulnerability [60].

Midlife alcohol consumption [61] shows a non-linear association with later cognitive outcomes, with both abstinence and frequent drinking associated with increased risk of mild cognitive impairment in old age [62]. Importantly, the risk of dementia increased with higher alcohol consumption only among carriers of the apolipoprotein E ε4 allele, indicating that alcohol acts as a conditional risk modifier whose detrimental effects on neurodegeneration are unmasked in the presence of genetic susceptibility rather than exerting uniform effects across populations [63].

In a large prospective cohort of primary care attenders aged 75 years and older, light-to-moderate alcohol consumption was associated with a lower observed incidence of both overall dementia and AD over a three-year follow-up period. However, alcohol use was strongly correlated with favorable sociodemographic and mental health characteristics, including higher education, absence of depression, and not living alone, leading the authors to caution that the inverse association may reflect survivor effects and residual confounding rather than a direct neuroprotective effect of alcohol [64].

In a detailed clinic neuropsychological study of institutionalized elderly individuals, alcohol-related dementia accounted for approximately one quarter of dementia cases and was characterized by younger age at onset, milder cognitive impairment, and relative stability or improvement following abstinence compared with AD. Despite similar levels of global cognitive dysfunction at presentation, patients with alcohol-related dementia showed less progression over time and lacked definitive Alzheimer-type neuropathological criteria, supporting alcohol-related dementia as a distinct, partially reversible condition rather than a direct manifestation of AD pathology [65].

Alcohol use disorder is associated with a substantially increased risk of AD in older adults, independent of major demographic and medical comorbidities. In a large matched cohort derived from U.S. insurance claims data, individuals with alcohol use disorder exhibited a nearly twofold higher hazard of developing AD compared with controls across sex and racial subgroups, supporting alcohol use disorder as a strong disease-modifying factor that increases neurodegenerative vulnerability rather than a benign lifestyle exposure [66].

Chronic alcohol dependence is associated with mild but broad cognitive impairment affecting attention, working memory, executive function, and verbal fluency, even in the absence of overt dementia [67]. Comparative neuropsychological profiling demonstrates that alcohol-dependent individuals share frontal and executive deficits of similar magnitude to patients with mild AD while exhibiting relatively less severe episodic memory impairment, indicating overlapping but distinct cognitive vulnerability patterns between alcohol-related brain damage and AD [68].

Chronic and heavy alcohol exposure induces a sustained neuroinflammatory response that directly impairs mitochondrial bioenergetics and dynamics in the aging brain, thereby increasing vulnerability to AD. Alcohol activates microglia through innate immune pathways and promotes the excessive production of pro-inflammatory cytokines and reactive oxygen species, which together disrupt oxidative phosphorylation, mitochondrial integrity, and neuronal energy homeostasis, establishing a permissive environment for neurodegenerative progression [20].

Chronic alcohol consumption induced intestinal microbiota dysbiosis and compromises gut barrier integrity in a transgenic AD mouse model, resulting in increased intestinal permeability and endotoxemia. Although these alcohol-induced gut alterations did not uniformly exacerbate amyloid or tau pathology, they revealed a genotype- and sex-dependent susceptibility of the microbiota–gut–brain axis, highlighting intestinal and metabolic pathways as conditional modulators of AD-related vulnerability rather than direct drivers of classical neuropathology [69].

In cognitively normal adults, the relationship between alcohol consumption and brain structure varies according to genetic vulnerability to AD. Neuroimaging analyses show that alcohol intake interacts with AD polygenic risk to influence cortical thickness in regions affected early in disease progression, indicating that alcohol-related brain effects are conditional and modified by underlying genetic susceptibility rather than uniformly beneficial or harmful across populations [70].

In a long-term longitudinal study of cognitively normal individuals and patients with early AD followed for up to two decades, baseline daily alcohol consumption was not associated with increased risk of AD onset nor with a faster rate of cognitive decline after diagnosis. Rates of change on comprehensive psychometric measures were comparable between daily drinkers and less frequent drinkers, indicating that alcohol use, as assessed at study entry, did not measurably influence the longitudinal clinical course of AD in this cohort [71].

Chronic alcohol exposure induces a cascade of metabolic and cellular stress responses in the central nervous system, prominently involving the disruption of homocysteine metabolism, oxidative stress, and endoplasmic reticulum (ER) stress, all of which contribute to neuronal injury. Alcohol interferes with the methionine and transsulfuration pathways, leading to hyperhomocysteinemia, reduced activity of cystathionine β-synthase and cystathionine γ-lyase, and impaired sulfur amino acid metabolism. These alterations promote redox imbalance, mitochondrial dysfunction, and activation of the unfolded protein response, mechanisms that are increasingly recognized in neurodegenerative conditions, including AD [72].

Short-term moderate chronic alcohol exposure induces early molecular changes in the brain that resemble metabolic features of AD, even in the absence of robust amyloid accumulation. Experimental evidence shows that alcohol feeding rapidly increases frontal lobe phospho-tau levels while dysregulating insulin-related metabolic hormones and energy homeostasis, supporting the concept that alcohol establishes a permissive metabolic and neuroendocrine environment that may contribute to later neurodegenerative vulnerability rather than directly driving classical Alzheimer’s pathology [73].

Chronic intermittent alcohol consumption across the lifespan produces sex- and genotype-specific effects in a transgenic AD rat model, with females showing greater vulnerability to cognitive and affective disturbances than males. Notably, alcohol history selectively modulated hippocampal inflammatory and AD-related gene expression, particularly under inflammatory challenge, indicating that alcohol acts as a conditional modifier of neurodegenerative and immune pathways rather than as a uniform driver of Alzheimer’s pathology [74].

Chronic heavy alcohol exposure disrupts liver–brain amyloid homeostasis by downregulating hepatic low-density lipoprotein receptor-related protein-1 (LRP-1), a key mediator of peripheral amyloid-β clearance. In transgenic AD mice, alcohol-induced reduction in hepatic LRP-1 was associated with increased brain amyloid-β accumulation, impaired microglial plaque association, blood–brain barrier alterations, and behavioral changes, highlighting the importance of liver–brain crosstalk in alcohol-dependent exacerbation of AD pathology [75]. Long-term ethanol-treated neural cultures exhibit suppressed basal oxidative stress but exaggerated inflammatory and apoptotic responses under superimposed hypoxic challenge, indicating that alcohol promotes a fragile state of stress tolerance that may transiently mask damage while ultimately lowering neuronal resilience to neurodegenerative insults [76]. Experimental data from primary rat glial cell cultures demonstrate that ethanol induces a non-linear, hormetic inflammatory response associated with oxidative stress and redox imbalance [77].

Intramitochondrial interaction between amyloid-β and amyloid-β-binding alcohol dehydrogenase (ABAD) is a critical mediator of early mitochondrial dysfunction in AD [78]. Experimental inhibition of the ABAD–amyloid-β interaction restores mitochondrial respiratory chain activity, reduces oxidative stress, enhances mitochondrial amyloid-β degradation, and improves spatial learning and memory in transgenic AD mice, identifying ABAD-dependent mitochondrial toxicity as a key non-plaque mechanism contributing to neurodegeneration [79].

A history of repeated alcohol intoxication [80] accelerates the emergence of cognitive impairment and induces transcriptional changes consistent with advanced AD progression in genetically susceptible mice. In a triple-transgenic AD mouse model, chronic alcohol intake was shown to worsen hippocampal-dependent memory, increase amyloid-β ratios and tau pathology in specific brain regions, and disrupt Akt/mTOR signaling, indicating that even moderate alcohol exposure can amplify neurodegenerative processes in susceptible brains [81]. Single-nucleus RNA sequencing of the prefrontal cortex revealed that alcohol exposure shifts neuronal, glial, and vascular gene expression profiles toward those observed in older AD models, particularly involving neuroinflammatory, mitochondrial, and synaptic pathways, autophagy, and blood–brain barrier maintenance, indicating that excessive alcohol intake promotes molecular signatures of disease progression rather than initiating classical pathology de novo [82,83].

In contrast, studies examining light-to-moderate alcohol consumption typically report neutral or inverse associations with dementia risk [84]. Protective associations are often confined to narrow intake ranges, commonly less than one standard drink per day, and disappear or reverse at slightly higher levels [85]. In patients with newly diagnosed mild AD, baseline alcohol consumption was not associated with accelerated cognitive decline but showed a non-linear relationship with short-term mortality. Specifically, individuals reporting moderate alcohol intake (approximately 2–3 units per day) exhibited lower all-cause mortality over a 36-month follow-up compared with both abstinent patients and those with higher intake, highlighting the complexity of alcohol–outcome relationships in clinically established AD and underscoring the influence of disease stage and competing health factors [86].

Molecular dynamics simulations indicate that alcohols alter the local solvation environment of the amyloid-β_1–42_ peptide and destabilize key intramolecular salt bridges that regulate monomer conformation and toxicity. Changes in alcohol concentration and hydrophobicity modify water structuring around residues Asp23, Lys28, and Ala42, promoting conformations associated with increased amyloid toxicity and aggregation propensity, thereby suggesting a physicochemical mechanism by which alcohol exposure can influence early amyloid misfolding processes [87].

For decades, observational studies have suggested that light-to-moderate alcohol consumption, particularly within Mediterranean dietary patterns, might confer cognitive or survival benefits in older adults [88]. However, more recent large-scale longitudinal and genetically informed analyses challenge this interpretation, indicating that apparent protective effects are largely explained by lifestyle clustering, socioeconomic factors, and reverse causation, rather than a direct neuroprotective action of alcohol itself. This emerging evidence marks a substantial shift in the conceptual framework of alcohol and dementia research, reframing moderate alcohol intake as context-dependent and potentially harmful rather than inherently protective [23].

Early population-based prospective studies reported that light-to-moderate alcohol consumption was associated with a lower incidence of dementia, including AD, particularly at intake levels of one to three drinks per day. In the Rotterdam Study, this inverse association was most evident for vascular dementia and appeared independent of beverage type, reinforcing the long-standing hypothesis that moderate alcohol intake might confer cognitive benefit through vascular or metabolic mechanisms [89].

Dose–response meta-analytic evidence indicates that the association between alcohol consumption and dementia risk is non-linear and highly sensitive to exposure definition and analytical approach [90,91]. While very low levels of alcohol intake have been associated with slightly reduced relative risk compared with abstinence, dementia risk increases at higher consumption levels [92]. Dose–response associations between alcohol consumption and dementia risk in observational studies are highly susceptible to bias arising from competing mortality and incomplete ascertainment of dementia status [93].

When dementia is assessed only at discrete follow-up visits and death is treated as simple censoring, relative risk estimates may be substantially distorted, particularly for exposures such as alcohol that are strongly associated with mortality. These methodological limitations can artificially generate or exaggerate apparent protective effects of moderate alcohol intake, underscoring the need for illness–death models and cautious interpretation of non-linear alcohol–dementia associations [93].

Careful clinicopathological analyses indicate that a distinct “primary alcoholic dementia” attributable solely to the direct neurotoxic effects of alcohol lacks a well-defined and consistent neuropathological substrate. Instead, most cases of dementia in chronic alcohol users can be explained by secondary conditions such as Wernicke–Korsakoff syndrome, nutritional deficiencies, hepatic encephalopathy, or cerebrovascular disease, with radiological and cognitive abnormalities often showing partial reversibility after abstinence, arguing against alcohol as an independent cause of irreversible Alzheimer-type neurodegeneration [94].

## 3. Mechanistic Neuropathology and Biomarker Findings

Neurocognitive disorders related to alcohol use and AD share substantial overlap in cognitive deficits and brain alterations, particularly within memory-related networks. Multimodal neuroimaging demonstrates that both conditions commonly affect the medial temporal lobe, thalamus, and posteromedial cortex, while alcohol-related neurocognitive disorder is characterized by more extensive white-matter and cerebellar involvement, underscoring shared vulnerability pathways alongside distinct patterns of alcohol-specific brain injury [95]. Translationally, these partially overlapping neuroanatomical signatures suggest convergent network-level vulnerability, while the disproportionate white-matter and cerebellar involvement in alcohol-related disease is consistent with myelin/oligodendroglial susceptibility, microvascular injury, and metabolic stress phenotypes.

Prolonged exposure to high concentrations of ethanol followed by withdrawal markedly sensitized hippocampal CA1 pyramidal neurons to otherwise non-toxic amyloid-β insult [96]. In organotypic hippocampal slice cultures, ethanol withdrawal produced selective CA1 neurodegeneration and dramatically amplified amyloid-β–induced cytotoxicity through NMDA receptor-dependent excitotoxic mechanisms, indicating that alcohol exposure lowers the threshold for AD-related neuronal injury rather than directly inducing amyloid pathology [97]. This “threshold-lowering” effect is mechanistically consistent with glutamatergic dysregulation, calcium overload, mitochondrial permeability transition, and downstream activation of proteases and caspase-mediated apoptosis, and is therefore more compatible with vulnerability amplification than with amyloid initiation.

Alcohol use interacts with aging and the APOE ε4 genotype to promote AD-related vulnerability through immune-mediated pathways rather than through direct amyloid-driven mechanisms [98]. Evidence indicates that chronic and heavy alcohol exposure enhances microbial translocation, accelerates inflammaging and senescence-associated secretory phenotypes, and amplifies neuroinflammatory signaling, particularly in genetically susceptible individuals, thereby acting as a disease-modifying factor that lowers the threshold for AD development [99]. At the biomarker level, this framework predicts parallel changes in systemic inflammatory mediators, microglial activation markers, and endothelial dysfunction indices, which may precede or exceed changes in classical amyloid/tau biomarkers.

Genetic variation in alcohol-metabolizing enzymes modulates vulnerability to AD through pathways linked to oxidative stress rather than through direct effects on alcohol consumption itself. In a Taiwanese population, plasma levels of alcohol dehydrogenase 1C (ADH1C) were significantly higher in patients with AD, and a sex-specific association between an ADH1C polymorphism and AD risk was observed in a pilot cohort, underscoring the relevance of alcohol metabolism-related redox burden as a conditional modifier of neurodegenerative susceptibility [100]. Mechanistically, altered alcohol/aldehyde handling is expected to increase reactive aldehyde load, lipid peroxidation adduct formation, and mitochondrial redox imbalance—molecular events that can be indexed by biomarkers such as 4-hydroxynonenal–protein adducts and related oxidative damage signatures.

Integrated transcriptomic and machine-learning analyses reveal that AD and alcohol dependence share a common molecular signature enriched in ferroptosis-related pathways. Differentially expressed genes involved in iron handling, lipid peroxidation, and immune regulation, including CYBB, STEAP3, and ACSL4, were identified as central nodes linking alcohol dependence with AD-associated metabolic and inflammatory dysregulation, supporting ferroptosis as a convergent vulnerability mechanism rather than a disease-specific driver [101]. This convergence provides an actionable translational hypothesis: alcohol exposure may expand the labile iron pool and sensitize lipid membranes to peroxidation, while inflammatory NADPH oxidase activity (CYBB) amplifies reactive species generation—thereby coupling immune activation with iron-dependent oxidative injury.

Reduced activity of mitochondrial aldehyde dehydrogenase 2 (ALDH2), whether due to genetic polymorphisms or chronic alcohol exposure, promotes the accumulation of toxic aldehydes such as 4-hydroxynonenal and formaldehyde in the brain, leading to oxidative stress, mitochondrial dysfunction, neuroinflammation, and blood–brain barrier impairment. Experimental models demonstrate that ALDH2 deficiency alone can recapitulate AD-like cognitive decline and pathology, while alcohol exposure further exacerbates these processes, indicating that impaired aldehyde detoxification is a critical modifier of neurodegenerative vulnerability [102]. Here, the mechanistic chain is particularly biomarker-accessible: aldehyde accumulation (e.g., 4-HNE), mitochondrial injury markers, and BBB permeability indices provide intermediate phenotypes bridging exposure to cognitive outcome.

Chronic moderate alcohol intake, delivered under nutritionally controlled conditions, exacerbates AD-relevant brain changes in aged transgenic mice without uniformly increasing classical amyloid pathology. In Tg2576 mice, alcohol feeding reduced survival and spatial memory performance and increased endogenous insoluble amyloid-β levels, while these brain alterations correlated with markers of mild liver injury, highlighting a liver–brain axis through which alcohol can modulate neurodegenerative vulnerability independently of overt changes in human amyloid burden [103]. This supports a systemic-to-central mechanism in which hepatic dysfunction, altered peripheral clearance pathways (including LRP-1-dependent transport), and circulating inflammatory mediators indirectly shape central amyloid handling, microglial responses, and neurovascular integrity.

Excessive alcohol intake increases reactive oxygen species production, disrupts mitochondrial respiratory function, and activates innate immune pathways involving microglia, astrocytes [104], and Toll-like receptor signaling [105,106], processes that have been implicated in the development and progression of AD and other neurodegenerative disorders [106]. Oxidative stress-driven mitochondrial dysfunction is a central contributor to AD-related neurotoxicity [107], leading to synaptic failure, neuronal apoptosis, and cognitive impairment. In this context, oxidative injury is not a generic phenomenon but a mechanistically structured cascade linking mitochondrial electron transport disruption, redox-sensitive signaling, and glial-mediated cytokine amplification. Experimental models demonstrate that restoration of mitochondrial antioxidant capacity and redox balance attenuates amyloid-β-induced neuronal damage, improves cholinergic function, and rescues learning and memory deficits, underscoring the importance of mitochondrial resilience in modulating neurodegenerative vulnerability [108]. Consistent translational readouts include markers of lipid peroxidation, glutathione depletion, and altered mitochondrial enzyme activity, which may function as mechanistic intermediates between alcohol exposure and cognitive phenotype.

Hypoxic and ischemic stress in the central nervous system activates redox-sensitive transcriptional programs, notably hypoxia-inducible factor-1α and NF-κB signaling, leading to mitochondrial dysfunction, oxidative stress, and sustained neuroinflammatory responses [77]. These mechanisms interact closely with sulfur amino acid metabolism and redox homeostasis, indicating that disruption of endogenous protective pathways—such as H_2_S-dependent signaling—can lower neuronal resilience and facilitate neurodegenerative vulnerability relevant to AD progression [109]. This intersection is conceptually important for the present review because H_2_S-dependent persulfidation can modulate NF-κB activation status, mitochondrial efficiency, and endothelial homeostasis, positioning sulfur biology as a resilience layer within hypoxia/inflammation-driven injury cascades.

In an amyloid-β-induced mouse model of AD, sesquiterpene compounds with antioxidant and anti-inflammatory properties significantly attenuated memory impairment and reduced oxidative stress, as evidenced by decreased lipid peroxidation and restoration of glutathione levels. These findings support the concept that modulation of redox homeostasis and mitochondrial resilience, rather than direct targeting of amyloid burden alone, can substantially influence cognitive outcomes in AD [110].

In a streptozotocin-induced sporadic AD rat model, perillyl alcohol significantly attenuated oxidative–nitrosative stress and cholinergic dysfunction while improving spatial learning and memory. Combined treatment with polyvinyl alcohol-coated selenium nanoparticles and mesenchymal stem cells markedly improved cognitive performance while restoring hippocampal antioxidant capacity and brain-derived neurotrophic factor levels [111]. These effects were accompanied by the restoration of endogenous antioxidant defenses, normalization of acetylcholinesterase activity, and improvement of mitochondrial redox balance, indicating that targeting metabolic and oxidative pathways can substantially modify neurodegenerative phenotypes independently of direct amyloid-centric interventions [112]. Importantly, these studies exemplify how mechanistic biomarkers (antioxidant enzymes, BDNF, cholinergic markers, oxidative–nitrosative indices) can be used to map intervention effects onto defined biological pathways.

Alcohol extracts from *Ganoderma lucidum* were shown to delay AD-like progression in transgenic mouse models by restoring dysregulated DNA methylation patterns and improving cognitive performance. Treatment normalized global CpG methylation levels, upregulated key DNA methyltransferases, reduced neuronal loss, and decreased intracellular amyloid-β accumulation, highlighting epigenetic and metabolic modulation as important mechanisms influencing neurodegenerative vulnerability [113]. This evidence underscores that alcohol-related exposures and alcohol-derived bioactives can engage mechanistically distinct pathways (e.g., epigenetic regulation), reinforcing the need for a pluralistic model in which redox, immune, metabolic, and epigenetic modules interact.

Alcohol use disorder and AD share convergent pathological mechanisms centered on the sustained activation of innate immune pathways, particularly microglial reactivity mediated by TLR4 and NLRP3 inflammasome signaling. Experimental and clinical evidence indicates that chronic alcohol exposure induces a persistent neuroinflammatory state characterized by cytokine release, oxidative stress, synaptic loss, and blood–brain barrier disruption, processes that overlap substantially with immune-driven mechanisms known to accelerate AD progression rather than initiate amyloid pathology de novo [114]. From a translational standpoint, this positions innate immune biomarkers (e.g., cytokine panels, inflammasome-related mediators, and glial activation signatures) as candidate mechanistic readouts linking alcohol exposure to neurodegenerative acceleration.

Nonpharmacological interventions that enhance systemic redox balance, mitochondrial efficiency, and neurovascular function—such as physical exercise and lifestyle modulation—consistently target biological domains implicated in AD vulnerability [115]. Such interventions are mechanistically informative because they simultaneously modulate multiple convergent pathways—redox buffering, mitochondrial biogenesis, endothelial function, and inflammatory tone—thereby operationalizing the concept of resilience networks that may be compromised by chronic alcohol exposure.

## 4. Alcohol-Induced Disruption and Hydrogen Sulfide Signaling in AD

Lower dietary methionine intake is associated with improved cognitive function and reduced risk of mild cognitive impairment, effects that are mechanistically linked to activation of the cystathionine β-synthase (CBS)/H_2_S pathway. In APP/PS1 AD model mice, methionine restriction enhanced brain H_2_S levels, alleviated oxidative stress and mitochondrial dysfunction, and improved synaptic integrity and memory performance, supporting a role for sulfur amino acid metabolism and H_2_S signaling in modulating neurodegenerative vulnerability [116]. At the pathway level, methionine restriction shifts sulfur flux toward transsulfuration, increasing CBS-dependent conversion of homocysteine + serine → cystathionine and promoting H_2_S-generating reactions from cysteine/homocysteine substrates, thereby reinforcing redox buffering capacity.

H_2_S exhibits a dose- and context-dependent “Janus-faced” role in neurodegenerative disease, acting as a cytoprotective regulator of redox balance, mitochondrial function, and neuroinflammation at physiological levels, while becoming deleterious when deficient or excessively accumulated. Dysregulation of H_2_S homeostasis amplifies oxidative stress, impairs mitochondrial respiration, and disrupts inflammatory control, positioning altered sulfide signaling as a permissive factor that lowers neuronal resilience and facilitates AD-related pathological cascades rather than serving as a singular causal driver [117]. This state dependence reflects the balance between (i) enzymatic synthesis (CBS/CSE/3-MST), (ii) storage and transfer in sulfane sulfur pools and protein persulfides (R–SSH), and (iii) mitochondrial oxidation pathways, which jointly determine cellular sulfide tone.

H_2_S regulates oxidative stress, inflammatory signaling, and extracellular matrix remodeling through transcriptional control, redox modulation, and post-translational S-sulfhydration of key proteins. By inhibiting NF-κB activation, reducing reactive oxygen species, and modulating matrix metalloproteinases and CD147/EMMPRIN activity, H_2_S preserves vascular and cellular integrity under chronic inflammatory conditions, highlighting its role as a protective biosignaling pathway whose dysregulation may lower tissue resilience in systemic and neurodegenerative diseases [118]. Mechanistically, persulfidation (R–SH → R–SSH) is a reversible cysteine modification that can reprogram redox-sensitive signaling nodes, including transcriptional regulators (e.g., Nrf2-linked antioxidant programs) and inflammatory mediators (e.g., NF-κB pathway components), thereby translating sulfide bioavailability into systems-level stress responses.

Altered H_2_S metabolism is associated with structural brain changes and cognitive dysfunction in AD and related dementias. Elevated circulating sulfide species, particularly bound and acid-labile sulfides, correlate with reduced cortical thickness, increased ventricular volume, and impaired white-matter integrity, supporting a link between dysregulated sulfide signaling, neurovascular dysfunction, and accelerated neurodegeneration [119]. Importantly, circulating sulfide species may reflect compensatory systemic shifts or altered sulfide partitioning, and therefore should be interpreted as indicators of dysregulated sulfide handling rather than simple “more vs. less H_2_S” effects.

Experimental and exogenous augmentation of H_2_S signaling ameliorates cognitive deficits and neuronal loss in AD models by enhancing endogenous sulfide-producing enzymes and activating antioxidant defense pathways. In APP/PS1 transgenic mice, administration of an H_2_S donor improved spatial learning and memory, reduced amyloid burden, preserved hippocampal neurons, and restored redox homeostasis through activation of the Nrf2-dependent antioxidant response, underscoring the capacity of H_2_S to modulate neurodegenerative vulnerability at multiple mechanistic levels [120]. These findings are consistent with a mechanistic sequence in which increased sulfide tone enhances antioxidant gene expression, preserves mitochondrial function, and stabilizes synaptic physiology, resulting in improved cognitive endpoints.

Endogenous H_2_S levels and the activity of H_2_S-synthesizing enzymes decline progressively with age in APP/PS1 transgenic mice, paralleling the emergence of amyloid pathology and cognitive impairment. Restoration of physiological H_2_S signaling through administration of an H_2_S donor improved spatial memory and shifted amyloid precursor protein processing away from the amyloidogenic pathway by downregulating β- and γ-secretase activity while enhancing α-secretase-mediated cleavage, thereby reducing brain amyloid burden [121]. This suggests that H_2_S can influence proteostatic processing of APP through redox-sensitive modulation of enzymatic activity and/or membrane microenvironment, thereby coupling metabolic redox state to amyloidogenic potential.

Microbiota-derived H_2_S exerts concentration-dependent effects on gut and blood–brain barrier integrity, glial activation, and neuroinflammatory signaling, thereby influencing neurodegenerative vulnerability [122]. Physiological H_2_S levels support barrier function and immune homeostasis, whereas dysbiosis-associated overproduction promotes systemic inflammation, microglial activation, and neuronal dysfunction, linking altered H_2_S homeostasis to AD progression through gut–brain axis mechanisms rather than direct amyloid initiation [123]. In translational terms, this implies that gut-derived sulfide, endotoxemia, and inflammatory mediators act as coupled signals that can modulate BBB permeability and central immune tone.

Exposure to sulfurous mineral waters and therapeutic mud produces measurable systemic anti-inflammatory and antioxidant effects, including significant reductions in circulating inflammatory markers and enhancement of endogenous antioxidant defenses. In vivo and in vitro evidence demonstrates that sulfur-rich natural treatments modulate redox balance and cellular resilience without inducing toxicity, supporting the concept that H_2_S-related pathways can influence oxidative stress and inflammatory states [124] relevant to neurodegenerative vulnerability [125]. This supports the broader principle that low-dose, context-appropriate sulfur signaling can engage adaptive redox and inflammatory programs.

In peripheral blood mononuclear cells from patients with AD, exposure to sulfurous water rich in H_2_S significantly reduced oxidative DNA damage and preserved cell viability under pro-oxidant conditions. These protective effects were comparable to or greater than those achieved with classical antioxidants and highlight the capacity of H_2_S to enhance cellular resistance to oxidative stress in AD-related systemic pathology [126]. Although peripheral, such findings provide mechanistic plausibility for sulfide-dependent cytoprotection via redox buffering and stress-response modulation.

Binge and chronic alcohol exposure induce the release of extracellular cold-inducible RNA-binding protein (eCIRP) from activated microglia, which acts as a damage-associated molecular pattern to amplify neuroinflammation and promote AD-related tau pathology. eCIRP signaling through receptors such as TLR4 and IL-6Rα activates downstream inflammatory and kinase pathways, linking alcohol-induced immune activation to tau hyperphosphorylation, neuronal dysfunction, and cognitive impairment independently of direct amyloid plaque accumulation [127]. This pathway is mechanistically relevant to sulfide biology because inflammatory signaling and redox imbalance can reciprocally suppress sulfide-dependent protective signaling, creating a feed-forward loop between innate immune activation, oxidative stress, and impaired resilience.

**Figure 3:**
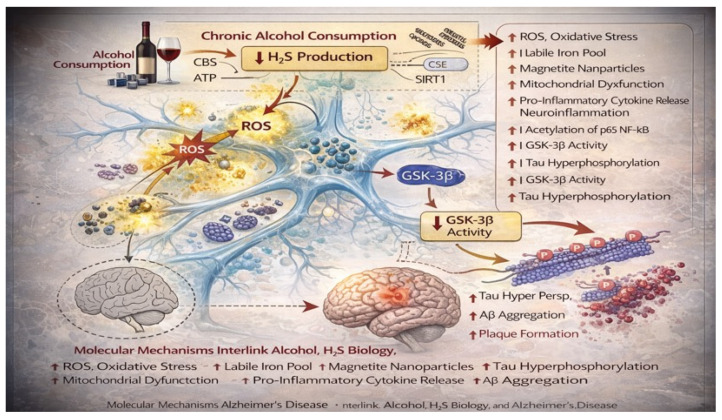
Alcohol-induced dysregulation of H_2_S signaling as a mechanistic amplifier of AD–related pathology. Multilevel molecular cascade through which chronic alcohol consumption perturbs H_2_S biology and promotes neurodegenerative processes relevant to AD. The decline in H_2_S bioavailability weakens its physiological roles in maintaining redox homeostasis, mitochondrial efficiency, and anti-inflammatory signaling. As a consequence, neurons exhibit increased reactive oxygen species (ROS) generation, expansion of the labile iron pool, accumulation of magnetite nanoparticles, and mitochondrial dysfunction, all of which synergistically amplify oxidative stress and metabolic failure. At the signaling level, alcohol-induced H_2_S deficiency facilitates activation of pro-inflammatory transcriptional pathways, including enhanced acetylation and activation of NF-κB, leading to increased release of pro-inflammatory cytokines and sustained neuroinflammation. In parallel, redox imbalance and inflammatory signaling converge on glycogen synthase kinase-3β (GSK-3β), whose activity is aberrantly increased under conditions of reduced sulfhydration.

Alcohol–H_2_S mechanistic interface (Figure 3). A key translational gap in the field is direct human evidence that alcohol exposure reduces brain H_2_S; however, several mechanistically coherent nodes link alcohol metabolism to impaired sulfide signaling: (i) disruption of sulfur amino acid metabolism with hyperhomocysteinemia and altered transsulfuration flux (CBS/CSE-dependent), (ii) increased reactive aldehydes and ROS that consume redox buffering capacity and impair persulfidation-dependent regulation, (iii) mitochondrial injury that perturbs sulfide oxidation capacity and bioenergetic coupling, and (iv) innate immune activation (e.g., TLR4/NF-κB axis) that amplifies inflammatory stress normally restrained by sulfide-dependent signaling. Together, these nodes support a model in which alcohol shifts the system from adaptive sulfide-mediated resilience toward vulnerability. Rather than reducing total sulfide levels uniformly, chronic alcohol exposure may preferentially impair effective H_2_S signaling by disrupting the balance between sulfide production, storage, and utilization. Altered transsulfuration flux, oxidative consumption of persulfide pools, and mitochondrial dysfunction may shift sulfide from signaling-competent forms (e.g., protein persulfides and sulfane sulfur) toward inactive or maladaptive species. Under this hypothesis, conventional measurements of circulating or total sulfide may fail to capture biologically meaningful deficits in sulfide-dependent signaling, explaining the current lack of consistent human evidence despite strong mechanistic plausibility.

H_2_S plays a dual, concentration-dependent role in AD by modulating redox homeostasis, mitochondrial function, neuroinflammation, vascular integrity, and tau phosphorylation through sulfhydration-dependent signaling mechanisms. Dysregulation of H_2_S metabolism, including altered activity of cystathionine β-synthase and 3-mercaptopyruvate sulfurtransferase, is associated with vascular dysfunction, blood–brain barrier impairment, enhanced GSK-3β activity, and tau hyperphosphorylation, positioning disturbed H_2_S signaling as a permissive factor that amplifies neurodegenerative vulnerability rather than acting as a singular causal driver of Alzheimer’s pathology [128].

H_2_S regulates neuronal resilience by modulating mitochondrial enzymes, redox-sensitive transcription factors, and inflammatory signaling cascades, and both insufficient and excessive sulfide disrupt cellular homeostasis. Altered H_2_S metabolism amplifies oxidative stress, compromises mitochondrial respiration, and weakens blood–brain barrier integrity, thereby facilitating neurodegenerative vulnerability in AD without acting as a primary amyloid-initiating factor [129].

H_2_S acts as a redox-sensitive cytoprotective gasotransmitter that preserves mitochondrial bioenergetics, limits reactive oxygen species generation, and suppresses chronic inflammatory signaling in aging tissues. Dysregulation of H_2_S synthesis or signaling shifts this balance toward oxidative stress, endothelial dysfunction, and impaired cellular resilience, providing a biologically plausible mechanism through which systemic stressors—such as chronic alcohol exposure—may exacerbate neurovascular and neurodegenerative vulnerability relevant to AD [130]. The alcohol–H_2_S–AD axis is best framed as a conditional interaction: alcohol increases stress load, and disrupted sulfide-dependent signaling reduces adaptive capacity, together accelerating vulnerability trajectories.

## 5. Discussion

The present review integrates epidemiological, experimental, and mechanistic evidence to examine alcohol-induced dysregulation of H_2_S signaling and connections to AD pathophysiology. Rather than advancing a direct causal model, the synthesis supports a pluralistic and systems-oriented interpretation in which the working hypothesis is that alcohol does not primarily accelerate neurodegeneration through direct enhancement of amyloid-β or tau pathology but instead acts as a disease-modifying stressor that disrupts endogenous protective signaling systems.

Alcohol-related dementia [131] exhibits a neuropsychological profile distinct from AD [132], characterized by impaired free recall with relatively preserved recognition memory and inconsistent deficits in procedural learning. Comparative testing demonstrated that, unlike AD, alcohol-related dementia does not conform cleanly to either a cortical or subcortical dementia pattern but instead reflects a mixed phenotype, suggesting diffuse brain vulnerability driven by chronic alcohol exposure rather than classical AD pathology [133].

Longitudinal studies in older adults regarding long-term alcohol use shows that it contributes to cognitive impairment and dementia risk primarily as a compounding factor rather than a direct cause of AD [134]. Evidence indicates that heavy alcohol consumption increases vulnerability to cognitive decline through interacting mechanisms such as oxidative stress, nutritional deficiency, cerebrovascular injury, and elevated homocysteine levels, thereby acting along the causal pathway to dementia in conjunction with aging, genetic susceptibility, and comorbid disease rather than inducing a distinct alcohol-specific Alzheimer’s pathology [135]. These convergent mechanisms preferentially affect metabolic, vascular, and inflammatory homeostasis, domains that critically condition neuronal survival under aging-related stress.

In this context, H_2_S emerges as a central integrative pathway linking alcohol exposure to redox imbalance, mitochondrial dysfunction, neuroinflammation, and vascular injury, all of which are increasingly recognized as critical determinants of AD vulnerability and progression.

A population-based case–control study found that alcohol consumption prior to disease onset was associated with a lower observed risk of AD, particularly among women and individuals who never smoked, while tobacco use showed no independent association. Importantly, the authors emphasized that these associations were highly dependent on gender, smoking status, and exposure history, underscoring the complexity of alcohol–AD relationships and the potential influence of interaction effects and methodological bias rather than a uniform protective effect [136].

From the perspective of previous epidemiological studies, the results help reconcile long-standing inconsistencies in the alcohol–dementia literature. While heavy alcohol use and alcohol use disorder consistently increase dementia risk—particularly for early-onset disease—light-to-moderate alcohol consumption has often been associated with neutral or seemingly protective effects. The present synthesis suggests that these divergent findings are unlikely to reflect intrinsic neuroprotection by alcohol. Instead, they may arise from methodological biases and from conditional biological effects, whereby low-level exposure does not overwhelm endogenous protective systems, including H_2_S-mediated redox and vascular regulation. In contrast, chronic or heavy alcohol exposure appears to exceed the buffering capacity of these systems, leading to a functional collapse of H_2_S signaling and subsequent amplification of neurodegenerative processes. This interpretation aligns epidemiological heterogeneity with a mechanistic threshold model of resilience failure.

Amyloid-β can bind to mitochondrial alcohol dehydrogenase, forming the Aβ-binding alcohol dehydrogenase (ABAD) complex, which disrupts mitochondrial redox homeostasis and promotes the generation of reactive oxygen species and toxic aldehydes, including formaldehyde [137]. This interaction impairs mitochondrial respiration, amplifies oxidative stress, and establishes a self-reinforcing cycle in which aldehyde accumulation accelerates amyloid aggregation, tau hyperphosphorylation, and neuronal injury, thereby contributing to AD pathogenesis [138]. Notably, this mechanism further supports a mitochondrial-centric vulnerability model rather than a plaque-centric initiation paradigm.

Excessive alcohol consumption is proposed to accelerate AD progression by disrupting central serotonergic circuitry, particularly within the dorsal raphe nucleus, a brainstem region affected early by tau pathology. Alcohol-induced serotonergic dysfunction, together with neuroinflammation and proteostatic stress, may contribute to early non-cognitive symptoms and facilitate the spread of tau pathology independently of amyloid-β deposition, highlighting a vulnerability pathway through which alcohol can modify AD trajectories [139]. This pathway exemplifies how neurotransmitter systems intersect with inflammatory and metabolic stress to shape disease progression.

Variants of the serotonin transporter gene (SLC6A4) appear to exert a shared modulatory influence on vulnerability to both alcohol dependence and AD rather than conferring disorder-specific risk [140]. Analyses across independent clinical samples indicate that while SLC6A4 polymorphisms show minimal association with major psychiatric disorders, several variants are more frequently observed in individuals with alcohol abuse and AD, suggesting a common neurobiological susceptibility pathway linking serotonergic regulation, addictive behaviors, and neurodegenerative processes [140]. Such genetic intersections further support a conditional, network-based model of risk rather than disease-specific causation.

Future research should prioritize the direct investigation of H_2_S signaling in human studies, including measurement of sulfide pools, enzyme activity, and persulfidation status in individuals with varying patterns of alcohol exposure and cognitive impairment. Longitudinal studies integrating alcohol exposure, sulfur metabolism, genetic risk factors such as APOE ε4, and multimodal biomarkers will be essential to clarify temporal relationships and susceptibility windows. Experimental work should further explore whether restoration of H_2_S signaling—through pharmacological donors, modulation of sulfur amino acid metabolism, or lifestyle interventions—can mitigate alcohol-related acceleration of neurodegenerative processes. Such approaches may offer novel therapeutic and preventive strategies not only for AD but also for alcohol-related cognitive disorders more broadly.

H_2_S signaling exhibits state-dependent effects, where identical concentrations can be protective or toxic depending on redox state, mitochondrial function, and cellular context. Also, H_2_S acts through redox-sensitive modulation of protein networks, rather than through single receptor–ligand interactions, allowing for distributed and rapid system-level responses. Persulfidation introduces reversible, low-energy modifications that can fine-tune protein conformation and signaling cascades, conceptually resembling threshold-sensitive or probabilistic regulatory systems rather than deterministic linear pathways.

The relationship between alcohol consumption and dementia risk remains highly sensitive to study design, exposure definition, and choice of reference group. While observational cohorts have often reported lower dementia risk among light-to-moderate drinkers compared with abstainers, careful methodological analyses indicate that these findings are strongly influenced by abstainer misclassification, competing mortality, and residual confounding, leading to uncertainty about causality and reinforcing the need for mechanistic frameworks to interpret alcohol–dementia associations [141].

Multidisciplinary interventions in neurodegenerative disorders, including AD, are associated with small-to-moderate but clinically meaningful improvements in quality of life, particularly when programs are intensive, sustained, and include psychological components. Meta-analytic evidence indicates that longer intervention duration and integration of psychosocial care enhance patient-reported outcomes, underscoring the importance of systemic, resilience-oriented approaches alongside biological mechanisms in modifying neurodegenerative vulnerability [142].

Experimental and translational evidence indicates that physiological stressors such as physical exercise upregulate H_2_S-producing pathways, while exogenous low-dose sulfur exposure can modulate these signals within a hormetic framework, supporting the concept that H_2_S-dependent biosignaling contributes to systemic resilience against oxidative and inflammatory stress relevant to neurodegenerative vulnerability [143]. These observations further reinforce the view of H_2_S as an adaptive mediator whose preservation may slow vulnerability trajectories rather than reverse established pathology.

## 6. Conclusions

This review synthesizes multidisciplinary evidence to propose H_2_S signaling as a central but non-exclusive and context-dependent, mechanistic link between alcohol exposure and AD vulnerability. The findings indicate that alcohol does not consistently promote AD through the direct amplification of amyloid-β or tau pathology. Instead, alcohol appears to function primarily as a disease-modifying stressor that disrupts endogenous protective systems governing redox balance, mitochondrial integrity, neuroinflammatory control, and neurovascular function. Within this framework, dysregulation of H_2_S signaling provides a biologically coherent explanation for the heterogeneous and often conflicting observations reported in the alcohol–dementia literature.

The review highlights that chronic and heavy alcohol exposure perturbs sulfur amino acid metabolism, promotes hyperhomocysteinemia, and interferes with the enzymatic pathways responsible for endogenous H_2_S production. These alterations plausibly result in a functional H_2_S-deficient state, lowering neuronal resilience to aging-related and AD-related stressors. This model is consistent with experimental evidence demonstrating the neuroprotective role of H_2_S and with human studies showing that alcohol-related cognitive impairment frequently occurs in the absence of classical Alzheimer’s pathology and may exhibit partial reversibility with abstinence. Such reversibility supports a vulnerability–resilience paradigm rather than an irreversible lesion-based model.

By integrating epidemiological, experimental, and mechanistic data, this review advances a systems-level perspective in which alcohol-induced disruption of H_2_S signaling accelerates neurodegenerative processes without necessarily initiating canonical AD lesions. This conceptual shift has important implications for risk stratification, prevention, and therapeutic development. It emphasizes the value of targeting resilience-modulating pathways—such as sulfide-dependent redox, metabolic, and vascular regulation—alongside classical disease markers. Preserving or restoring H_2_S signaling may represent a novel strategy to mitigate the alcohol-related acceleration of cognitive decline and potentially slow the progression of AD.

## Data Availability

No new data were created or analyzed in this study. Data sharing is not applicable to this article.
